# Obituary: Peter J. Quesenberry, MD (1938–2025)

**DOI:** 10.1002/jev2.70279

**Published:** 2026-04-14

**Authors:** Giovanni Camussi, Yong Song Gho, Andrew F. Hill, Jan Lötvall, Clotilde Théry

**Affiliations:** ^1^ University of Torino Turin Piedmont Italy; ^2^ POSTECH Pohang Gyeongbuk South Korea; ^3^ Victoria University Melbourne Victoria Australia; ^4^ University of Gothenburg Gothenburg Sweden; ^5^ Institut Curie Paris France


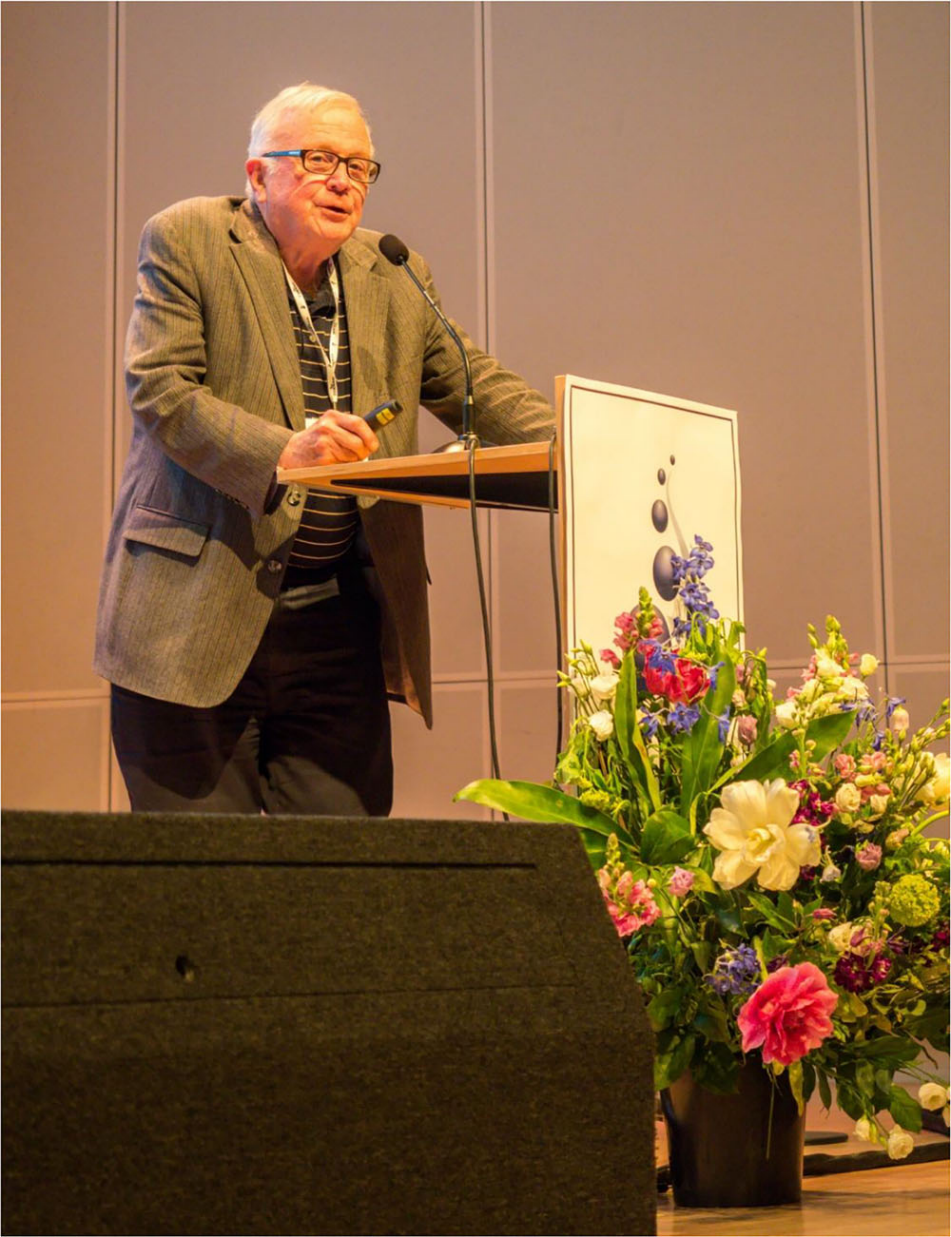


Peter J. Quesenberry, MD, a visionary physician‐scientist whose work bridged hematology, stem cell biology, and extracellular vesicle (EV) research, passed away on November 13, 2025, at the age of 87. Professor Quesenberry was a leading figure in experimental hematology for more than five decades and played a formative role in establishing EV biology as a rigorous and translational scientific discipline.

Trained as a hematologist–oncologist, he made seminal contributions to understanding hematopoietic stem cell heterogeneity, cell‐cycle–dependent stem cell behaviour, and bone marrow plasticity. In the later stages of his career, he was among the first to integrate EVs into stem cell biology, advancing the concept that EVs are potent mediators of cell fate, tissue repair, and phenotypic modulation. This work helped drive the translational shift in the field by demonstrating the biological and clinical relevance of EVs.

Professor Quesenberry's impact on the EV field extended well beyond his laboratory. Peter was elected to the Executive Board of the International Society of Extracellular Vesicles (ISEV) as a Member at Large for two terms from 2012 (the first ISEV board) to 2016. Additionally, He, along with Clotilde Thery and Yong Song Gho, were the first Editor‐in‐Chiefs of the *Journal of Extracellular Vesicles* (from 2012 to 2019) and played a central role in shaping the journal's scientific scope, editorial standards, and global visibility (Théry et al. 2019). In recognition of this service, he was jointly awarded the ISEV Special Achievement Award in 2014, acknowledging his foundational contributions to both EV science and the society itself.

A prolific scholar, Quesenberry authored more than 400 peer‐reviewed publications and over 100 book chapters, many of which influenced successive generations of hematologists, stem cell biologists, and EV researchers. Equally important was his role as a mentor and intellectual catalyst, being known for challenging prevailing dogma, encouraging conceptual risk‐taking, and fostering interdisciplinary thinking. His legacy is reflected not only in his discoveries but also in the scientists and clinician‐investigators he trained, many of whom continue to shape the EV and stem cell fields today.

Picture: Peter Quesenberry presenting at the first ISEV Annual Meeting in Gothenburg, 2012. Photo credit: Jan Kwarnmark.
